# Management of Melasma: Laser and Other Therapies—Review Study

**DOI:** 10.3390/jcm13051468

**Published:** 2024-03-03

**Authors:** Badea Jiryis, Ohad Toledano, Emily Avitan-Hersh, Ziad Khamaysi

**Affiliations:** 1 Department of Dermatology, Rambam Health Care Campus, Haifa 3109601, Israel; badi.jiryis@campus.technion.ac.il (B.J.); e_avitan@rambam.health.gov.il (E.A.-H.); 2 Bruce Rappaport Faculty of Medicine, Technion Institute of Technology, Haifa 3525433, Israel; 3 Alma Lasers Ltd., Caesarea 38900, Israel; ohadtoledano@gmail.com

**Keywords:** melasma, laser, treatment

## Abstract

Melasma is a commonly occurring pigmented skin condition that can significantly affect one’s appearance, described as symmetric hyperpigmentation that presents as irregular brown to gray-brown macules on various facial areas, such as the cheeks, forehead, nasal bridge, and upper lip, along with the mandible and upper arms. Due to its complex pathogenesis and recurrent nature, melasma management is challenging and the outcomes following treatment are not always deemed satisfactory. Solely treating hyperpigmentation may prove ineffective unless paired with regenerative techniques and photoprotection, since one of the main reasons for recurrence is sun exposure. Hence, the treatment protocol starts with addressing risk factors, implementing stringent UV protection, and then treatment using different strategies, like applying topical treatments, employing chemical peels, laser and light therapies, microneedling, and systemic therapy. This review aims to provide a summary of the effectiveness and safety of the frequently employed laser and light therapies for treating melasma, focusing on laser therapy as a treatment for melasma.

## 1. Introduction

Melasma, a common dermatological condition characterized by the presence of hyperpigmented patches and macules, typically manifests on the facial skin. This condition predominantly impacts adult females, particularly those with Fitzpatrick skin phototypes III to V [1].

So far, the exact cause of melasma remains not fully understood. Nevertheless, it is suggested that chronic exposure to ultraviolet (UV) radiation, hormonal factors in females, such as pregnancy and oral contraceptive medication, and a genetic predisposition are all factors that have been proposed to be involved in the development of melasma [2,3,4].

The treatment of melasma continues to pose a challenge due to the limited clinical effectiveness, high recurrence rates, and the occurrence of adverse reactions associated with many of the treatments. The management of melasma typically commences with preventive measures involving sun protection followed by a range of treatments that encompass topical applications, oral therapies, chemical peels, microneedling, laser procedures, and light treatments [4,5]. The most utilized topical agent is hydroquinone, but it frequently results in irritant and allergic contact dermatitis [6]. Oral tranexamic acid (TA) is known to cause menstrual irregularities and may increase the risk of deep venous thrombosis [7]. Chemical peels, commonly used as an adjunctive therapy, often come with side effects like burning and peeling of the skin [8].

Laser treatments, which have arisen as a newer method, have been increasingly used in recent years. The mechanism of light selectively damaging pigment particles in pigment cells, offers precision in targeting different skin chromophores without damaging adjacent normal tissues, so it takes effect quickly and does not produce scars [4].

However, it is important to note that some studies have reported adverse effects, including erythema, a burning sensation, post-inflammatory hyperpigmentation (PIH), hypopigmentation, and the possibility of melasma recurrence [9,10,11]. Additionally, some studies have suggested that combining laser therapy with other treatments does not yield significantly better results compared to laser therapy alone [12].

Despite advances in technology, our understanding of the effectiveness and safety of laser treatments for melasma remains incomplete. This study aims to review the current treatments focusing on laser therapy as a treatment for melasma.

## 2. Management

### 2.1. Overview

The initial approach to treating melasma in patients generally involves addressing or removing risk factors, implementing rigorous protection against ultraviolet sun exposure, and using topical formulations designed to lighten the skin. While topical treatments may provide temporary improvement, the condition frequently recurs. Treatment principles aim to inhibit melanin synthesis pathways, reduce melanosome transfer from melanocytes to keratinocytes, and expedite pathways for melanin removal. This review aims to compile and present recent studies that have investigated treatment of melasma using energy-based devices.

### 2.2. Preventative Practices

Photoprotection is the cornerstone of the prevention and treatment of melasma [13]. Exposure to sunlight has been observed to trigger and worsen hyperpigmentation in individuals with melasma. Adopting habits like minimizing sun exposure duration, donning protective clothing to limit exposure to the sun, and diligently applying sunscreen can aid in the management of melasma. Sun protection topicals take precedence and are often employed as a supportive measure alongside other melasma treatments, as both ultraviolet (UV) and visible light can trigger prolonged hyperpigmentation across all skin types [14,15]. Due to the underlying mechanisms of melasma, depending solely on UVA/UVB protection is insufficient. Sunscreens designed for this condition should provide broad-spectrum coverage, including UVA1 and HEVL, with a high SPF for robust defense. Additionally, they should be aesthetically pleasing for regular use [13].

### 2.3. Topical Treatment

The mainstay of melasma treatment has historically been topical therapies, with the most utilized topical agent being hydroquinone 2–5% [6]. Other topical agents, including azelaic acid, kojic acid, and tranexamic acid, have demonstrated a substantial reduction in melasma and should be taken into consideration before opting for chemical peels or laser therapy [16]. The most effective formulation to date has been Kligman’s formula, which consists of a cream base containing hydroquinone, tretinoin, and dexamethasone. This formulation has been shown to lead to improvements or even complete clearance in as many as 60–80% of treated patients and is still regarded as the gold standard in melasma treatment [17,18,19].

#### 2.3.1. Hydroquinone

For quite some time, hydroquinone (HQ), a hydroxyphenolic compound, has held its reputation as the primary medication for treating melasma. Its effectiveness stems from its capability to hinder the transformation of DOPA into melanin by attaching to copper, thereby inhibiting tyrosinase [5]. Moreover, HQ has demonstrated involvement in breaking down melanosomes and melanocytes [20]. Research findings indicated that a combination cream containing 4% HQ, 0.05% tretinoin, and 0.01% fluocinolone acetonide demonstrated slightly superior effectiveness compared to using 4% HQ alone or in combination with one other ingredient [5,21,22,23]. The frequently reported adverse effect is irritant contact dermatitis, marked by symptoms such as erythema, a burning sensation, pruritus, and scaling. This has been identified as a prevalent short-term adverse reaction, observed in as many as 70% of patients, primarily associated with HQ doses of 4% or higher, although it can manifest across different concentrations of HQ [6,24]. In 2006, the FDA proposed the discontinuation of over-the-counter (OTC) hydroquinone at concentrations of 1–2%, primarily due to concerns regarding significant absorption rates, documented cases of exogenous ochronosis in human studies, and findings of potential carcinogenicity in animal studies. This ban was officially enforced in 2020 with the passage of the CARES Act. Presently, hydroquinone products are only available through FDA-approved new drug applications or in formulations prescribed for individual patients. This regulatory shift stemmed from reported instances of exogenous ochronosis, even with low concentrations of HQ (1–2%), compounded by the wide availability of unregulated OTC hydroquinone [25,26,27].

#### 2.3.2. Retinoids

Retinoids aid in the metabolism and renewal of keratinocytes, which helps decrease the transfer of melanosomes and speeds up the loss of melanin. Additionally, they enhance the penetration of other topical medications through the skin’s outer layer (transepidermal penetration) [28,29]. Tretinoin (0.05–0.1%) continues to be a popular retinoid used successfully in treating melasma, often as a standalone treatment. However, visible clinical improvement typically requires a minimum of 24 weeks of consistent application [1]. Studies evaluating the efficacy of tretinoin on Caucasians and African Americans treated with ATRA 0.1% cream versus placebo daily for 40 weeks, showed a statistically significant improvement in severity compared to placebo in the Caucasians group [30], though the African American group showed only marginal statistical significance [31]. Various retinoids, such as adapalene, tazarotene, and topical isotretinoin, have been employed in the treatment of melasma alongside hydroquinone [32,33].

#### 2.3.3. Corticosteroids

Corticosteroids are directly involved in UV-B-induced melanogenesis by inhibiting substances like prostaglandins and cytokines—such as endothelin 1 and granulocyte macrophage colony-stimulating factor (GM-CSF)—that contribute to increasing melanin production [34]. The application of a potent or super potent corticosteroid alone topically has shown effective therapeutic results [35]. However, topical corticosteroids are not commonly employed as a standalone treatment for melasma due to their potential adverse effects on the skin, including skin atrophy, facial hypertrichosis, acne-like eruptions, telangiectasias, rosacea, and perioral dermatitis [5]. Corticosteroids like hydrocortisone, dexamethasone, mometasone furoate, fluocinolone acetonide, and fluticasone continue to be favored in triple combination therapies for treating melasma [36].

#### 2.3.4. Triple Combination Therapy

Kligman-illis introduced the initial triple combination in 1975, which contained hydroquinone 5%, tretinoin 0.1%, and dexamethasone 0.1%. The combination of hydroquinone with a retinoid and a corticosteroid continues to be the most extensively studied approach for treating melasma due to its enhanced tolerability and efficacy [37]. The efficacy of this combination stems from the synergistic action of its individual components. The visible benefits typically appear after approximately three weeks of twice-daily application. Tretinoin aids by preventing the oxidation of hydroquinone and enhancing the penetration of other agents into the skin. Concurrently, the topical corticosteroid decreases cellular metabolism, inhibits melanin synthesis, and reduces potential irritation caused by the other two components [37]. In a recent Cochrane review, triple combination therapy emerged as the most effective topical treatment for melasma overall [38]. The most frequently encountered side effects include erythema and scaling. Less common effects comprise dryness, a burning sensation, and pruritus [21]. The TC cream has not only proven to be effective but has also demonstrated its safety when used daily for eight weeks. This is followed by a maintenance phase involving intermittent application (either twice weekly or a tapering regimen) for six months [39].

#### 2.3.5. Chemical Peeling

Chemical peels stimulate skin regeneration and tissue remodeling by applying exfoliative agents, and they are commonly used for the treatment of melasma. The remodeling process can be categorized into three levels: superficial, which impacts the epidermis through the papillary dermis; medium, which involves the papillary to the upper reticular dermis; and deep, which penetrates through the mid-reticular dermis [40,41].

Managing melasma in individuals with dark skin is difficult due to a greater likelihood of post-inflammatory hyperpigmentation when compared to those with lighter skin [42]. Chemical peels are a recognized treatment for melasma, as they remove superficial lesions and stimulate tissue regeneration. However, it is advisable to avoid deep or medium-depth peels in dark-skinned patients to prevent hyperpigmentation [43].

#### 2.3.6. Active Substances of Natural Origin and Plant Ferments

Natural substances with skin-whitening properties have emerged as potential treatments for melasma, offering alternatives to synthetic chemicals and their associated side effects. These extracts, sourced from plants, marine organisms, bacteria, and fungi, possess promising therapeutic qualities [44]. Among the plant-derived skin-whitening agents is the Korean red ginseng (KRG) that shows good tolerability and 100 beneficial effects for melasma [45]. Ocean-derived agents like Endarachne binghamiae, Schizymenia dubyi, Ecklonia cava (EC), and Sargassum silquastrum (SS) mostly suppresses melanin production by inhibiting the enzymatic activity of tyrosinase [46]. Kojic acid, which is widely used in combination with other substances and proven to be effective as melasma treatment, is naturally derived from fungal metabolites produced by species of Acetobacter, Aspergillus, and Penicillium [47]. Fermented plants have garnered attention as another treatment option due to their skin-lightening effects. Notably, fermented aloe vera leaf skin is highlighted as a cost-effective and low-risk natural ingredient that could potentially replace synthetic and chemical skin-lightening components with fewer side effects [48].

#### 2.3.7. Other Antioxidants

Antioxidants are widely used substances for multiple dermatologic indications including melasma. Among these substances are amino fruits, vitamin C (ascorbic acid), vitamin E, vitamin B3 (Niacinamide), phytic acid, polypodium leucotomos, silymarin and zinc are prominent examples. Amino fruit acid peels are carboxylated acidic amino acids which have been found to be less irritating and better tolerated than glycolic acid peels for melasma treatment [49]. Vitamin C has demonstrated efficacy in melasma treatment, particularly when used in combination therapy with Q-switched Nd:YAG laser to enhance its effects [50]. Niacinamide, or nicotinamide, is an amide of vitamin B3, is a safe and effective alone (compared with hydroquinone) [49], and as a combination therapy of 5% niacinamide, 3% TXA, and 1% kojic acid, there was significant decrease in the melanin index by week 12 as compared to both pre-treatment baseline and control [51]. Additionally, topical application of 10% zinc sulfate solution has been deemed safe and effective for treating melasma [52].

#### 2.3.8. Platelet-Rich Plasma

Platelet-rich plasma (PRP), a commonly utilized treatment in various dermatologic conditions, has emerged as a viable option for addressing melasma in recent years [53]. Its effectiveness in melasma treatment lies in its ability to inhibit melanin synthesis through delayed extracellular signal-regulated kinase activation. In a split-face prospective study involving 40 melasma cases, intradermal PRP application proved to be effective. Tranexamic acid (TXA) was intradermally injected into one side of the face at a concentration of 4 mg/mL, while PRP was injected into the opposite side. Each side received three treatment sessions at four-week intervals. Significant reductions in the melasma area and severity index (mMASI) score were observed on both sides after treatment (*p* < 0.001). However, the percentage of score reduction was notably higher on the PRP-treated side compared to the TXA-treated side [54]. Furthermore, a recent study indicated that combining topical 5% tranexamic acid in a liposome based cream and autologous PRP injection is more effective than tranexamic acid alone [55].

### 2.4. Systemic Treatment

Before prescribing systemic therapies, physicians should take the initiative to educate themselves and inform the patient about the appropriate dosage, potential side effects, and necessary precautions. Many systemic therapies can be used in melasma treatment, and orally administered *P. leucotomos* may provide significant therapeutic benefits for melasma, as it can be used as an adjuvant to topical treatment of melasma, particularly when used in conjunction with hydroquinone cream [56,57]. A randomized double-blind placebo-controlled clinical trial found oral polypodium leucotomos extract to be a safe and effective adjunctive treatment in combination with topical hydroquinone and sunscreen for melasma [58]. Oral tranexamic acid is increasingly being utilized due to its affordability and ease of prescription in general medical practice. A recent meta-analysis of randomized controlled trials, evaluating the use of oral tranexamic acid in adult melasma, demonstrated notable effectiveness and safety [59].

### 2.5. Microneedling

Microneedling, which is also referred to as percutaneous collagen induction therapy, is a minimally invasive procedure that entails the insertion of fine needles into the skin, often achieved through techniques such as stamping, needle rollers, or an electric-powered pen [60,61]. Microneedling has the potential to enhance transdermal drug delivery [62]. Additionally, the microinjuries that result from microneedling appear to activate genes related to epidermal differentiation and tissue remodeling when studied in vitro [63]. Recent studies prove microneedling combined with topical interventions to be an effective measure in melasma treatment [64,65]. In combination therapy with a 4% hydroquinone (HQ) cream and Q-switched Nd:YAG laser (QSNYL), microneedling has demonstrated superior results when compared to using topical or laser therapy alone [66,67,68]. The adverse effects associated with microneedling can range from temporary redness, a burning sensation, to pain, swelling, and bruising. It is important to note that the severity of these local reactions depends on the skills and technique of the operator [69,70].

### 2.6. Lasers and Light Based Therapy

#### 2.6.1. Overview

In 1983, Anderson and Parrish introduced the concept of laser therapy for treating cutaneous disorders. Their pioneering work highlighted the distinct thermal and absorptive characteristics of pigmented structures within the skin, rendering them suitable targets for precise destruction using specific wavelengths of radiation. Importantly, this approach spared the surrounding healthy tissue. Consequently, a wide range of targets, such as unwanted hair and tattoo ink, could be effectively removed with minimal impact on the surrounding normal skin [71]. Since that milestone, light and laser therapies have been found to be applicable in numerous dermatologic and cosmetic conditions, encompassing vascular birthmarks, telangiectasias, hypertrichosis, tattoo removal, pigmented lesions, solar lentigines, lentiginous nevi, café-au-lait macules, and melasma [72].

Laser therapy has emerged as a safe alternative option for treating melasma, particularly in cases where the more conventional methods involving topical creams and chemical peels have proven less effective. A wide array of laser therapies underwent extensive examination in numerous clinical trials, revealing a diverse spectrum of treatment effectiveness and potential adverse events. Typically, the results of these trials are assessed by medical professionals or based on changes in the Melasma Area Severity Index (MASI).

#### 2.6.2. Intense Pulsed Light (IPL)

Intense pulsed light (IPL) systems emit a range of different wavelengths between 515 and 1200 nanometer (nm). Within this spectrum of light, some of its wavelengths are selectively absorbed in the melanosomes. Another benefit of IPL technology is its multiple wavelengths that allow for the targeting of multiple layers of epidermal and dermal melasma simultaneously [73].

Based on meta-analysis from Jiarong Yi. In 2020 [74], IPL-based combination therapy for melasma can effectively reduce MASI scores and result in higher satisfaction among patients.

Typical side effects include mild erythema (skin redness) and a slight tingling sensation, which generally resolves within a day. In a few cases, some patients may encounter mild skin exfoliation due to the use of higher energy levels, but this typically heals without scarring in approximately one week [9,75].

#### 2.6.3. Low-Fluence Q-Switched 1064 nm Nd:YAG

Q-switched lasers are known for producing high-intensity laser beams with extremely short pulse durations. Their pulse velocity is about a million times faster than that of an IPL pulse. These lasers, specifically designed to target melanin, are available in various wavelengths, including ruby (694 nm), alexandrite (755 nm), and neodymium-doped yttrium aluminum garnet (Nd:YAG; 532 nm or 1064 nm) [76]. Among patients with melasma, the 1064 nm Q-switched neodymium-doped yttrium aluminum garnet (QSNY) laser is a popular choice, often used in combination with other treatment methods. However, the use of QSNY has been associated with adverse effects such as guttate hypopigmentation and melasma rebound. It is important to note that the application of high-fluence QSNYL in melasma treatment carries a risk of post-inflammatory pigmentation [77,78].

A new approach to using Q-switched lasers, referred to as low-fluence or subthermolytic Q-switched treatment, is becoming increasingly popular [73,79]. While the lasers used are the same, the fluences applied are lower compared to those traditionally used for treating other pigmented lesions. Low-fluence treatments primarily utilize the 1064 nm wavelength, which penetrates deeper into the dermis while sparing the epidermis to a greater extent. The rationale behind treating patients with melasma using subthermolytic low fluences is based on the concept that pigment disruption occurs via a photoacoustic mechanism, breaking down the pigment while preserving the keratinocytes and melanocytes from damage. Although there is typically some level of damage associated with subthermolytic Q-switched treatment, it is reported to be less than that observed with traditional photothermolytic approaches [80].

The optimal outcomes and reduced risk of recurrence are attained with this technique when it is combined with additional agents, including topical hydroquinone (HQ), azelaic acid, chemical peels like Jessner’s formula, glycolic acid (GA), and systemic treatment [81]. Moreover, clinical trials involving combination using other types of lasers, such as nonablative 1550 nm erbium-doped fractional photothermolysis, pulsed dye laser (PDL) and Er:YAG (2940 nm) laser, have been documented. In a meta-analysis by CHEN et al., it was suggested that the laser combination treatment approach yields superior results compared to monotherapy. This is attributed to the fact that combination therapy operates through diverse mechanisms in treating melasma, and their effects can be attributed to a synergistic superposition [82].

Notwithstanding the favorable therapeutic effects, the recurrence rate following these procedures remains notably high, making this treatment option not sufficiently effective. Low-fluence Q-switched laser therapy, especially using an Nd:YAG laser, has displayed promising initial results. However, it necessitates multiple treatments within a relatively short treatment interval (typically weekly) and is associated with exceptionally high 3-month recurrence rates ranging from 64% to 81%. The number of required treatments is generally higher compared to other light- and laser-based treatment modalities [83,84,85] and can affect patients’ compliance with the treatment. In terms of the LQSNY safety of the treatment, it has been demonstrated to induce adverse events, including erythema, transient burning, slight edema, microcrust, hyperpigmentation, hypopigmentation, and telangiectasia. Notably, guttate hypopigmentation has been reported in cases involving the combination of LQSNY with microneedling, glycolic acid, intense pulsed light (IPL), or hydroquinone, with incidence rates ranging from 5.5% to 13.6% [9,86].

#### 2.6.4. Non-Ablative Fractionated Resurfacing Lasers (NAFL)

Four laser wavelengths are used in this category including 1440 nm, 1540 nm, 1550 nm, and 1927 nm, namely within the short-wavelength infrared spectrum of light. Non-ablative fractionated lasers mechanism of action is in the form of photothermolysis. As laser energy targeted and absorbed by water-containing tissues, it induces selective thermal damage in the form of micro-beams with a diameter of less than 400 µm and can reach up to 1000 µm in depth. This process leads to the formation of microscopic thermal zones (MTZ) that impact collagen fibers and keratinocytes. Column-like necroses of keratinocytes, referred to as microscopic epidermal necrotic debris (MEND), develop within the epidermis. This formation enables keratinocytes and dermal components to migrate towards the stratum corneum, resulting in the removal of coagulated substances [87,88]. Immediately after the application of NAFLs on melasma lesions, melanin can be detected within MENDs within six days of treatment. Enlarged melanocytes, commonly observed in melasma lesions, remain reduced in number for up to three months after a single NAFL treatment. These findings suggest that MENDs act as “melanin shuttles”, contributing to the elimination of dermal and epidermal melanin [89,90].

The utilization of NAFLs appears to yield a more enduring clinical response compared to IPL or Q-switched laser treatments. This is particularly notable when patients undergo topical tyrosinase inhibitor treatment both before and after the laser surgery [81].

Common side effects include erythema, swelling and pain. These are typically short-term side effects lasting for 3 to 10 days, and overall, the treatment is regarded as having low downtime with a fast recovery process. Post-inflammatory hyperpigmentation (PIH) is a reported side effect in most clinical trials. The occurrence of PIH appears to be associated with the density of the microthermal zones and possibly arise as a byproduct of the heat generated during the treatment. Relapses often occur after NAFL treatments, usually noted between 3 and 6 months after treatment, which is better than IPL and Q-switched lasers, where recurrence can occur as early as 3 months after discontinuing the treatment [73].

#### 2.6.5. Ablative Fractionated Resurfacing Lasers (AFL)

Ablative fractionated resurfacing lasers have been documented for the treatment of melasma patients [91]. Lasers such as CO_2_ 10,600 nm and Er:YAG 2940 nm operate by having their light energy absorbed by water molecules in the tissue, resulting in tissue ablation [92]. Fractionated ablation creates microscopic injury zones, facilitating improved transepidermal drug delivery (TDD), extraction of transepidermal melanin, enhancement of dermal elastin tissue, neocollagenesis, basal membrane stabilization, and consequently, reduced interaction between keratinocytes and melanocytes with melanogenic dermal stimulators, leading to less noticeable pigmentation [86].

Adopting a fractionated approach minimizes epidermal injury, resulting in fewer side effects and a lower risk of dyspigmentation. However, it is not recommended to use AFLs for melasma treatment due to the significant occurrence of side effects and the likelihood of relapses. In cases where specialists do employ AFL treatments, they prefer a CO_2_ laser with very low fluency, usually as part of combination therapy [93]. In a recent prospective cohort comparative study involving 40 female participants, the efficacy of a single session of low-power fractional CO_2_ (10,600 nm) laser followed by Jessner’s solution peeling was compared to that of Jessner’s solution peeling alone. The study found that there was no significant difference between the two groups in terms of improvements in mMASI scores. Additionally, both groups were safe and effective for the treatment of melasma [94].

#### 2.6.6. Picosecond Lasers

Picosecond lasers represent cutting-edge technology capable of generating pulses within the picosecond timeframe. The use of extremely short laser pulse durations induces melanin fragmentation through a photoacoustic mechanism rather than a photothermal one. This laser technology is highly efficient in pigment removal without causing thermal damage to the surrounding tissues. Picosecond lasers are based on light emission at various wavelengths, mainly 532 nm (KTP), 755 nm (alexandrite), and 1064 nm (Nd:YAG) [81,95].

To date, there have only been a few studies exploring the use of picosecond lasers for the treatment of melasma, some recommended its use, while others presented association with high recurrence rates. Therefore, further studies are required to evaluate the efficacy of this novel technology [93,96].

### 2.7. Patient Counseling

Effective management of melasma is attributed, in part, to the strength of the patient–clinician relationship. When advising patients dealing with melasma, clinicians need to collaborate with patients to identify the most suitable treatment options and establish realistic expectations for the course of the condition. In a condition that disproportionately affects individuals of color, it is crucial to employ patient-centric language that is both culturally and racially sensitive. This ensures effective communication about available treatment options, the potential for side effects, and the proper use of treatments [97].

## 3. Conclusions

There are many treatment options for melasma, such as topical, systemic, or procedural options. In recent years, a multitude of laser and light devices has become available, with many being employed in the treatment of melasma. The emergence of post-treatment hyperpigmentation and elevated recurrence rates linked to Q-switched neodymium-doped yttrium aluminum garnet laser (QSNYL) has prompted the development of low-fluence Q-switched neodymium-doped yttrium aluminum garnet (LFQSNYL) and picosecond lasers, which exhibit reduced skin inflammation. Additionally, non-ablative fractional lasers have demonstrated efficacy, more than ablative fractional lasers, which are not recommended for melasma unlike the previous ones mentioned. The initial approach in treating melasma typically involves the use of topical lightening agents. The application of energy-based devices is generally reserved for patients with refractory or recurrent lesions or those seeking the swift resolution of their condition. However, using these devices as a monotherapy can sometimes worsen melasma and result in rebound lesions after the discontinuation of treatment. As a result, combining energy-based devices with topical lightening agents is recommended. This combination approach offers a higher response rate, a shorter duration of treatment, lower side effects (such as post-inflammatory hyperpigmentation), and a reduced recurrence rate. It is advisable to maintain topical therapy along with an increased number of sessions with energy-based devices to preserve treatment efficacy and minimize the likelihood of recurrence.
